# 5-(4-Chloro­phen­yl)-1-(2,4-dichloro­phen­yl)-4-methyl-*N*-(3-pyridylmeth­yl)-1*H*-pyrazole-3-carboxamide

**DOI:** 10.1107/S1600536809013609

**Published:** 2009-04-18

**Authors:** Xinhua He, Wu Zhong, Junhai Xiao, Zhibing Zheng, Song Li

**Affiliations:** a Beijing Institute of Pharmacology and Toxicology, Beijing 100850, People’s Republic of China

## Abstract

In the title compound, C_23_H_17_Cl_3_N_4_O, the benzene rings are oriented with respect to the pyrazole ring at dihedral angles of 39.9 (2) and 72.90 (13)° for the chloro­phenyl and di­chloro­phenyl rings, respectively. Inter­molecular C—H⋯N and C—H⋯Cl inter­actions are observed in the crystal packing.

## Related literature

For general background to pyrazole derivatives and their biological activity, see: Srivastava *et al.* (2008 ▶); LoVerme *et al.* (2009 ▶); Rinaldi-Carmona *et al.* (1994 ▶). For the synthesis, see: Li *et al.* (2007 ▶).
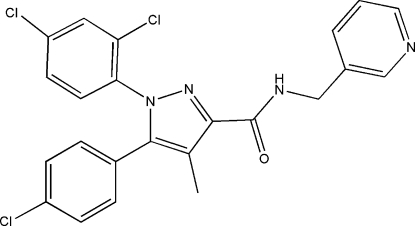


## Experimental

### 

#### Crystal data

C_23_H_17_Cl_3_N_4_O
*M* *_r_* = 471.76
Monoclinic, 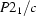

*a* = 9.0032 (4) Å
*b* = 20.1001 (8) Å
*c* = 11.4664 (5) Å
β = 92.003 (2)°
*V* = 2073.75 (15) Å^3^
*Z* = 4
Mo *K*α radiation
μ = 0.47 mm^−1^
*T* = 113 K
0.26 × 0.20 × 0.18 mm

#### Data collection

Rigaku Saturn CCD area-detector diffractometer
Absorption correction: multi-scan (*CrystalClear*; Rigaku/MSC, 2005 ▶) *T*_min_ = 0.888, *T*_max_ = 0.921
19184 measured reflections
4914 independent reflections
4152 reflections with *I* > 2σ(*I*)
*R*_int_ = 0.034

#### Refinement

*R*[*F* ^2^ > 2σ(*F* ^2^)] = 0.032
*wR*(*F* ^2^) = 0.085
*S* = 1.06
4914 reflections
285 parameters
H atoms treated by a mixture of independent and constrained refinement
Δρ_max_ = 0.75 e Å^−3^
Δρ_min_ = −0.38 e Å^−3^

### 

Data collection: *CrystalClear* (Rigaku/MSC, 2005 ▶); cell refinement: *CrystalClear*; data reduction: *CrystalClear*; program(s) used to solve structure: *SHELXS97* (Sheldrick, 2008 ▶); program(s) used to refine structure: *SHELXL97* (Sheldrick, 2008 ▶); molecular graphics: *SHELXTL* (Sheldrick, 2008 ▶); software used to prepare material for publication: *CrystalStructure* (Rigaku/MSC, 2004 ▶) and *publCIF* (Westrip, 2009 ▶).

## Supplementary Material

Crystal structure: contains datablocks I, global. DOI: 10.1107/S1600536809013609/ez2158sup1.cif

Structure factors: contains datablocks I. DOI: 10.1107/S1600536809013609/ez2158Isup2.hkl

Additional supplementary materials:  crystallographic information; 3D view; checkCIF report

## Figures and Tables

**Table 1:** Hydrogen-bond geometry (Å, °)

*D*—H⋯*A*	*D*—H	H⋯*A*	*D*⋯*A*	*D*—H⋯*A*
C17—H17⋯N4^i^	0.95	2.56	3.272 (2)	132
C7—H7⋯Cl2^ii^	0.95	2.84	3.5903 (15)	137

Symmetry codes: (i) 
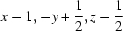
; (ii) 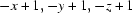.

**Fig. 1.**
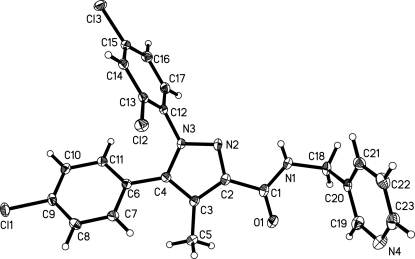
The molecular structure of the title compound, with the atom-numbering scheme and ellipsoids at the 30 % probability level.

**Fig. 2.**
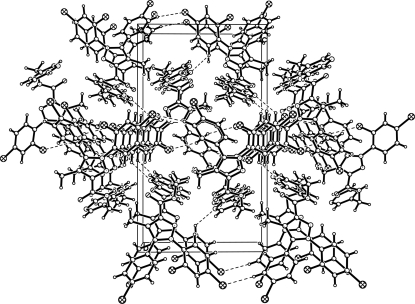
The crystal packing of the title compound. Hydrogen bonds are indicated by dashed lines.

## supplementary crystallographic information

### Comment

Pyrazole derivatives have been found to be a novel class of cannabinoid CB1 receptor antagonists (Srivastava *et al.*, 2008; LoVerme *et al.*, 2009; Rinaldi-Carmona M. *et al.*, 1994). The crystal structure of the title compound (IC50 =0.139nM at CB1) was analyzed by X-ray diffraction, for the purpose of studying its quantitative structure-activity relationship (QSAR).

In the molecule of the title compound (Fig. 1) the bond lengths and angles are generally within normal ranges. The benzene rings (C6—C11) and (C12—C17) are oriented at dihedral angles of 39.9 (2)° and 72.90 (13)°, respectively, with respect to the pyrazole ring.

In the crystal structure, the molecules are linked by intermolecular C17—H17···N4 and C7—H7···Cl2 interactions (Fig. 2).

### Experimental

The title compound was synthesized according to the procedure of Li *et al.*(2007). Colorless single crystals were obtained by slow evaporation of a solution in ehtyl acetate.

### Refinement

The H atoms linked to the C atoms were fixed geometrically and treated as riding with C—H = 0.95 Å (aromatic), 0.98Å (methyl), 0.99 Å (methylene) with *U*_iso_(H) =1.2–1.5Ueq(C). H atoms of the amino group were located in a difference Fourier map and refined freely.

### Crystal data

**Table:**

C_23_H_17_Cl_3_N_4_O	*F*(000) = 968
*M**_r_* = 471.76	*D*_x_ = 1.511 Mg m^−^^3^
Monoclinic, *P*2_1_/*c*	Mo *K*α radiation, λ = 0.71070 Å
*a* = 9.0032 (4) Å	Cell parameters from 4274 reflections
*b* = 20.1001 (8) Å	θ = 1.8–27.9°
*c* = 11.4664 (5) Å	µ = 0.47 mm^−^^1^
β = 92.003 (2)°	*T* = 113 K
*V* = 2073.75 (15) Å^3^	Block, colorless
*Z* = 4	0.26 × 0.20 × 0.18 mm

### Data collection

**Table:**

Rigaku Saturn CCD area-detector diffractometer	4914 independent reflections
Radiation source: rotating anode	4152 reflections with *I* > 2σ(*I*)
confocal	*R*_int_ = 0.034
Detector resolution: 7.31 pixels mm^-1^	θ_max_ = 27.9°, θ_min_ = 2.0°
ω and φ scans	*h* = −11→11
Absorption correction: multi-scan (*CrystalClear*; Rigaku/MSC, 2005)	*k* = −26→26
*T*_min_ = 0.888, *T*_max_ = 0.921	*l* = −15→15
19184 measured reflections	

### Refinement

**Table:**

Refinement on *F*^2^	Primary atom site location: structure-invariant direct methods
Least-squares matrix: full	Secondary atom site location: difference Fourier map
*R*[*F*^2^ > 2σ(*F*^2^)] = 0.032	Hydrogen site location: inferred from neighbouring sites
*wR*(*F*^2^) = 0.085	H atoms treated by a mixture of independent and constrained refinement
*S* = 1.06	*w* = 1/[σ^2^(*F*_o_^2^) + (0.0441*P*)^2^ + 0.5347*P*] where *P* = (*F*_o_^2^ + 2*F*_c_^2^)/3
4914 reflections	(Δ/σ)_max_ = 0.002
285 parameters	Δρ_max_ = 0.75 e Å^−^^3^
0 restraints	Δρ_min_ = −0.38 e Å^−^^3^

### Special details

**Table:**

Geometry. All e.s.d.'s (except the e.s.d. in the dihedral angle between two l.s. planes) are estimated using the full covariance matrix. The cell e.s.d.'s are taken into account individually in the estimation of e.s.d.'s in distances, angles and torsion angles; correlations between e.s.d.'s in cell parameters are only used when they are defined by crystal symmetry. An approximate (isotropic) treatment of cell e.s.d.'s is used for estimating e.s.d.'s involving l.s. planes.
Refinement. Refinement of *F*^2^ against ALL reflections. The weighted *R*-factor *wR* and goodness of fit *S* are based on *F*^2^, conventional *R*-factors *R* are based on *F*, with *F* set to zero for negative *F*^2^. The threshold expression of *F*^2^ > σ(*F*^2^) is used only for calculating *R*-factors(gt) *etc*. and is not relevant to the choice of reflections for refinement. *R*-factors based on *F*^2^ are statistically about twice as large as those based on *F*, and *R*- factors based on ALL data will be even larger.

### Fractional atomic coordinates and isotropic or equivalent isotropic displacement parameters (Å^2^)

**Table:**

	*x*	*y*	*z*	*U*_iso_*/*U*_eq_	
Cl1	0.01811 (4)	0.643967 (18)	0.56112 (3)	0.02233 (10)	
Cl2	0.52709 (4)	0.554267 (18)	0.22278 (3)	0.02014 (10)	
Cl3	0.15192 (4)	0.596113 (19)	−0.14473 (3)	0.02088 (10)	
O1	0.60926 (12)	0.24050 (6)	0.40372 (10)	0.0235 (3)	
N1	0.65174 (14)	0.25624 (6)	0.21143 (12)	0.0177 (3)	
H1	0.633 (2)	0.2770 (10)	0.1553 (17)	0.023 (5)*	
N2	0.47501 (14)	0.36428 (6)	0.20324 (11)	0.0158 (3)	
N3	0.38825 (14)	0.41730 (6)	0.22738 (11)	0.0149 (3)	
N4	1.09010 (16)	0.18709 (7)	0.40063 (13)	0.0255 (3)	
C1	0.59001 (16)	0.27258 (7)	0.31272 (14)	0.0160 (3)	
C2	0.49452 (15)	0.33347 (7)	0.30626 (13)	0.0145 (3)	
C3	0.41906 (16)	0.36537 (7)	0.39652 (13)	0.0142 (3)	
C4	0.35219 (16)	0.42006 (7)	0.34305 (13)	0.0138 (3)	
C5	0.40835 (18)	0.34400 (8)	0.52139 (13)	0.0203 (3)	
H5A	0.4938	0.3614	0.5672	0.030*	
H5B	0.4080	0.2953	0.5256	0.030*	
H5C	0.3163	0.3614	0.5529	0.030*	
C6	0.26488 (16)	0.47476 (7)	0.39330 (12)	0.0135 (3)	
C7	0.30933 (16)	0.49932 (8)	0.50303 (13)	0.0159 (3)	
H7	0.3927	0.4800	0.5432	0.019*	
C8	0.23420 (17)	0.55137 (7)	0.55469 (13)	0.0169 (3)	
H8	0.2662	0.5679	0.6290	0.020*	
C9	0.11196 (16)	0.57867 (7)	0.49587 (13)	0.0155 (3)	
C10	0.06310 (16)	0.55496 (7)	0.38774 (13)	0.0155 (3)	
H10	−0.0215	0.5740	0.3488	0.019*	
C11	0.13936 (16)	0.50292 (7)	0.33683 (13)	0.0144 (3)	
H11	0.1060	0.4863	0.2629	0.017*	
C12	0.33945 (16)	0.45989 (7)	0.13373 (12)	0.0143 (3)	
C13	0.39214 (16)	0.52489 (7)	0.12519 (13)	0.0144 (3)	
C14	0.33693 (16)	0.56739 (7)	0.03826 (13)	0.0154 (3)	
H14	0.3721	0.6118	0.0325	0.019*	
C15	0.22900 (16)	0.54305 (7)	−0.03979 (12)	0.0155 (3)	
C16	0.17985 (17)	0.47747 (7)	−0.03588 (13)	0.0181 (3)	
H16	0.1090	0.4613	−0.0923	0.022*	
C17	0.23618 (17)	0.43610 (8)	0.05187 (13)	0.0172 (3)	
H17	0.2037	0.3912	0.0557	0.021*	
C18	0.74791 (16)	0.19856 (7)	0.19942 (14)	0.0185 (3)	
H18A	0.7511	0.1867	0.1158	0.022*	
H18B	0.7040	0.1605	0.2408	0.022*	
C19	0.95085 (18)	0.18300 (8)	0.35444 (15)	0.0213 (3)	
H19	0.8786	0.1607	0.3985	0.026*	
C20	0.90543 (16)	0.20918 (7)	0.24668 (13)	0.0158 (3)	
C21	1.01070 (18)	0.24347 (8)	0.18393 (14)	0.0200 (3)	
H21	0.9844	0.2630	0.1106	0.024*	
C22	1.15446 (18)	0.24873 (8)	0.23013 (15)	0.0237 (3)	
H22	1.2281	0.2721	0.1892	0.028*	
C23	1.18931 (18)	0.21935 (8)	0.33690 (16)	0.0254 (4)	
H23	1.2889	0.2222	0.3666	0.031*	

### Atomic displacement parameters (Å^2^)

**Table:**

	*U*^11^	*U*^22^	*U*^33^	*U*^12^	*U*^13^	*U*^23^
Cl1	0.0286 (2)	0.01416 (17)	0.0247 (2)	0.00408 (14)	0.00882 (16)	−0.00229 (14)
Cl2	0.01844 (19)	0.02230 (19)	0.01936 (19)	−0.00254 (14)	−0.00415 (14)	−0.00143 (14)
Cl3	0.0230 (2)	0.02238 (19)	0.01701 (19)	0.00224 (15)	−0.00231 (14)	0.00519 (14)
O1	0.0245 (6)	0.0209 (6)	0.0252 (6)	0.0074 (5)	0.0007 (5)	0.0043 (5)
N1	0.0171 (6)	0.0151 (6)	0.0208 (7)	0.0055 (5)	−0.0006 (5)	0.0016 (5)
N2	0.0145 (6)	0.0130 (6)	0.0199 (7)	0.0034 (5)	0.0021 (5)	−0.0005 (5)
N3	0.0163 (6)	0.0135 (6)	0.0151 (6)	0.0048 (5)	0.0021 (5)	0.0009 (5)
N4	0.0232 (7)	0.0239 (7)	0.0290 (8)	0.0020 (6)	−0.0049 (6)	0.0042 (6)
C1	0.0110 (7)	0.0131 (7)	0.0239 (8)	−0.0007 (5)	−0.0010 (6)	−0.0006 (6)
C2	0.0108 (7)	0.0132 (6)	0.0194 (7)	−0.0009 (5)	−0.0002 (5)	0.0004 (6)
C3	0.0113 (7)	0.0139 (7)	0.0172 (7)	−0.0002 (5)	−0.0016 (5)	0.0003 (5)
C4	0.0127 (7)	0.0150 (7)	0.0136 (7)	−0.0003 (5)	0.0001 (5)	−0.0006 (5)
C5	0.0229 (8)	0.0208 (8)	0.0170 (8)	0.0047 (6)	−0.0015 (6)	0.0028 (6)
C6	0.0126 (7)	0.0136 (7)	0.0144 (7)	−0.0001 (5)	0.0024 (5)	0.0010 (5)
C7	0.0124 (7)	0.0202 (7)	0.0151 (7)	−0.0006 (6)	0.0000 (5)	0.0007 (6)
C8	0.0166 (7)	0.0186 (7)	0.0154 (7)	−0.0036 (6)	0.0016 (6)	−0.0025 (6)
C9	0.0165 (7)	0.0103 (6)	0.0201 (8)	−0.0010 (5)	0.0075 (6)	−0.0006 (5)
C10	0.0137 (7)	0.0139 (7)	0.0190 (7)	0.0010 (5)	0.0020 (6)	0.0033 (6)
C11	0.0148 (7)	0.0146 (7)	0.0139 (7)	−0.0006 (5)	0.0004 (5)	0.0004 (5)
C12	0.0156 (7)	0.0149 (7)	0.0128 (7)	0.0050 (5)	0.0037 (6)	0.0011 (5)
C13	0.0123 (7)	0.0173 (7)	0.0138 (7)	0.0017 (5)	0.0017 (5)	−0.0028 (5)
C14	0.0170 (7)	0.0142 (7)	0.0153 (7)	0.0006 (5)	0.0034 (6)	0.0002 (6)
C15	0.0168 (7)	0.0171 (7)	0.0127 (7)	0.0039 (6)	0.0024 (6)	0.0017 (6)
C16	0.0188 (7)	0.0198 (7)	0.0157 (7)	−0.0001 (6)	−0.0011 (6)	−0.0019 (6)
C17	0.0188 (7)	0.0142 (7)	0.0188 (8)	0.0004 (6)	0.0023 (6)	−0.0020 (6)
C18	0.0152 (7)	0.0142 (7)	0.0262 (8)	0.0031 (6)	−0.0004 (6)	−0.0041 (6)
C19	0.0194 (8)	0.0181 (7)	0.0265 (8)	−0.0005 (6)	0.0015 (6)	0.0041 (6)
C20	0.0154 (7)	0.0110 (6)	0.0212 (8)	0.0033 (5)	0.0012 (6)	−0.0037 (6)
C21	0.0238 (8)	0.0174 (7)	0.0190 (8)	0.0003 (6)	0.0029 (6)	−0.0015 (6)
C22	0.0198 (8)	0.0235 (8)	0.0284 (9)	−0.0049 (6)	0.0073 (7)	−0.0030 (7)
C23	0.0168 (8)	0.0247 (8)	0.0344 (10)	0.0002 (6)	−0.0033 (7)	−0.0044 (7)

### Geometric parameters (Å, °)

**Table:**

Cl1—C9	1.7437 (15)	C8—H8	0.9500
Cl2—C13	1.7268 (15)	C9—C10	1.386 (2)
Cl3—C15	1.7346 (15)	C10—C11	1.391 (2)
O1—C1	1.2336 (19)	C10—H10	0.9500
N1—C1	1.346 (2)	C11—H11	0.9500
N1—C18	1.4565 (18)	C12—C17	1.383 (2)
N1—H1	0.78 (2)	C12—C13	1.395 (2)
N2—C2	1.3399 (19)	C13—C14	1.391 (2)
N2—N3	1.3556 (16)	C14—C15	1.387 (2)
N3—C4	1.3776 (19)	C14—H14	0.9500
N3—C12	1.4303 (18)	C15—C16	1.392 (2)
N4—C23	1.341 (2)	C16—C17	1.388 (2)
N4—C19	1.346 (2)	C16—H16	0.9500
C1—C2	1.496 (2)	C17—H17	0.9500
C2—C3	1.412 (2)	C18—C20	1.515 (2)
C3—C4	1.386 (2)	C18—H18A	0.9900
C3—C5	1.501 (2)	C18—H18B	0.9900
C4—C6	1.480 (2)	C19—C20	1.391 (2)
C5—H5A	0.9800	C19—H19	0.9500
C5—H5B	0.9800	C20—C21	1.392 (2)
C5—H5C	0.9800	C21—C22	1.385 (2)
C6—C7	1.397 (2)	C21—H21	0.9500
C6—C11	1.4019 (19)	C22—C23	1.385 (2)
C7—C8	1.390 (2)	C22—H22	0.9500
C7—H7	0.9500	C23—H23	0.9500
C8—C9	1.384 (2)		
			
C1—N1—C18	122.73 (14)	C10—C11—H11	119.6
C1—N1—H1	119.9 (14)	C6—C11—H11	119.6
C18—N1—H1	117.3 (14)	C17—C12—C13	119.89 (13)
C2—N2—N3	104.00 (12)	C17—C12—N3	118.96 (13)
N2—N3—C4	112.65 (12)	C13—C12—N3	121.14 (13)
N2—N3—C12	118.77 (12)	C14—C13—C12	120.77 (13)
C4—N3—C12	128.47 (12)	C14—C13—Cl2	118.68 (11)
C23—N4—C19	116.36 (15)	C12—C13—Cl2	120.55 (11)
O1—C1—N1	123.54 (14)	C15—C14—C13	118.06 (13)
O1—C1—C2	122.20 (14)	C15—C14—H14	121.0
N1—C1—C2	114.26 (13)	C13—C14—H14	121.0
N2—C2—C3	112.63 (13)	C14—C15—C16	121.99 (13)
N2—C2—C1	118.63 (13)	C14—C15—Cl3	119.01 (11)
C3—C2—C1	128.74 (13)	C16—C15—Cl3	119.00 (12)
C4—C3—C2	104.40 (13)	C17—C16—C15	118.84 (14)
C4—C3—C5	127.40 (13)	C17—C16—H16	120.6
C2—C3—C5	128.16 (13)	C15—C16—H16	120.6
N3—C4—C3	106.29 (13)	C12—C17—C16	120.33 (14)
N3—C4—C6	123.44 (13)	C12—C17—H17	119.8
C3—C4—C6	130.20 (13)	C16—C17—H17	119.8
C3—C5—H5A	109.5	N1—C18—C20	113.96 (12)
C3—C5—H5B	109.5	N1—C18—H18A	108.8
H5A—C5—H5B	109.5	C20—C18—H18A	108.8
C3—C5—H5C	109.5	N1—C18—H18B	108.8
H5A—C5—H5C	109.5	C20—C18—H18B	108.8
H5B—C5—H5C	109.5	H18A—C18—H18B	107.7
C7—C6—C11	118.31 (13)	N4—C19—C20	124.54 (15)
C7—C6—C4	118.28 (13)	N4—C19—H19	117.7
C11—C6—C4	123.41 (13)	C20—C19—H19	117.7
C8—C7—C6	121.43 (14)	C19—C20—C21	117.51 (14)
C8—C7—H7	119.3	C19—C20—C18	120.44 (14)
C6—C7—H7	119.3	C21—C20—C18	122.03 (14)
C9—C8—C7	118.78 (14)	C22—C21—C20	118.94 (15)
C9—C8—H8	120.6	C22—C21—H21	120.5
C7—C8—H8	120.6	C20—C21—H21	120.5
C8—C9—C10	121.46 (13)	C21—C22—C23	119.02 (15)
C8—C9—Cl1	118.58 (12)	C21—C22—H22	120.5
C10—C9—Cl1	119.96 (11)	C23—C22—H22	120.5
C9—C10—C11	119.23 (13)	N4—C23—C22	123.58 (15)
C9—C10—H10	120.4	N4—C23—H23	118.2
C11—C10—H10	120.4	C22—C23—H23	118.2
C10—C11—C6	120.77 (13)		
			
C2—N2—N3—C4	−0.44 (16)	Cl1—C9—C10—C11	179.90 (11)
C2—N2—N3—C12	−177.01 (12)	C9—C10—C11—C6	−0.4 (2)
C18—N1—C1—O1	0.1 (2)	C7—C6—C11—C10	1.4 (2)
C18—N1—C1—C2	−179.79 (13)	C4—C6—C11—C10	−179.03 (14)
N3—N2—C2—C3	1.05 (16)	N2—N3—C12—C17	69.79 (18)
N3—N2—C2—C1	−178.45 (12)	C4—N3—C12—C17	−106.16 (17)
O1—C1—C2—N2	−177.73 (14)	N2—N3—C12—C13	−111.38 (15)
N1—C1—C2—N2	2.13 (19)	C4—N3—C12—C13	72.7 (2)
O1—C1—C2—C3	2.9 (2)	C17—C12—C13—C14	3.0 (2)
N1—C1—C2—C3	−177.28 (14)	N3—C12—C13—C14	−175.77 (13)
N2—C2—C3—C4	−1.25 (16)	C17—C12—C13—Cl2	−177.12 (11)
C1—C2—C3—C4	178.18 (14)	N3—C12—C13—Cl2	4.06 (19)
N2—C2—C3—C5	176.76 (14)	C12—C13—C14—C15	−0.4 (2)
C1—C2—C3—C5	−3.8 (2)	Cl2—C13—C14—C15	179.75 (11)
N2—N3—C4—C3	−0.31 (16)	C13—C14—C15—C16	−2.5 (2)
C12—N3—C4—C3	175.84 (14)	C13—C14—C15—Cl3	176.98 (11)
N2—N3—C4—C6	176.89 (13)	C14—C15—C16—C17	2.8 (2)
C12—N3—C4—C6	−7.0 (2)	Cl3—C15—C16—C17	−176.73 (11)
C2—C3—C4—N3	0.89 (15)	C13—C12—C17—C16	−2.8 (2)
C5—C3—C4—N3	−177.14 (14)	N3—C12—C17—C16	176.05 (13)
C2—C3—C4—C6	−176.05 (14)	C15—C16—C17—C12	−0.1 (2)
C5—C3—C4—C6	5.9 (3)	C1—N1—C18—C20	−78.52 (18)
N3—C4—C6—C7	−138.52 (15)	C23—N4—C19—C20	0.6 (2)
C3—C4—C6—C7	38.0 (2)	N4—C19—C20—C21	−1.8 (2)
N3—C4—C6—C11	41.9 (2)	N4—C19—C20—C18	176.56 (15)
C3—C4—C6—C11	−141.62 (16)	N1—C18—C20—C19	102.36 (17)
C11—C6—C7—C8	−1.5 (2)	N1—C18—C20—C21	−79.37 (18)
C4—C6—C7—C8	178.89 (13)	C19—C20—C21—C22	1.2 (2)
C6—C7—C8—C9	0.6 (2)	C18—C20—C21—C22	−177.11 (14)
C7—C8—C9—C10	0.4 (2)	C20—C21—C22—C23	0.4 (2)
C7—C8—C9—Cl1	180.00 (11)	C19—N4—C23—C22	1.3 (3)
C8—C9—C10—C11	−0.5 (2)	C21—C22—C23—N4	−1.8 (3)

### Hydrogen-bond geometry (Å, °)

**Table:**

*D*—H···*A*	*D*—H	H···*A*	*D*···*A*	*D*—H···*A*
C17—H17···N4^i^	0.95	2.56	3.272 (2)	132
C7—H7···Cl2^ii^	0.95	2.84	3.5903 (15)	137

Symmetry codes: (i) *x*−1, −*y*+1/2, *z*−1/2; (ii) −*x*+1, −*y*+1, −*z*+1.
